# Depressive symptom trajectories among general population during the COVID-19 pandemic in Iceland: a prospective cohort study (2020–2023)

**DOI:** 10.1136/bmjph-2024-001294

**Published:** 2024-11-04

**Authors:** Yue Wang, Arna Hauksdóttir, Edda Bjork Thordardottir, Fenfen Ge, Elísabet Unnur Gísladóttir, Jóhanna Jakobsdóttir, Kristjana Hrönn Ásbjörnsdóttir, Harpa Rúnarsdóttir, Anna Bára Unnarsdóttir, Ingibjörg Magnúsdóttir, Thorvardur Jon Love, Sigurdur Yngvi Kristinsson, Runolfur Palsson, Helga Zoega, Fang Fang, Gunnar Tómasson, Huan Song, Thor Aspelund, Unnur Valdimarsdottir

**Affiliations:** 1 Centre of Public Health Sciences, Faculty of Medicine, University of Iceland, Reykjavik, Iceland; 2 NCRR-The National Centre for Register-based Research, Aarhus University, Aarhus, Denmark; 3 Institute of Environmental Medicine, Karolinska Institutet, Stockholm, Sweden; 4 Department of Epidemiology, University of Washington, Seattle, Washington, USA; 5 Faculty of Medicine, University of Iceland, Reykjavik, Iceland; 6 Internal Medicine Services, Landspitali University Hospital, Reykjavik, Iceland; 7 Division of Hematology, Landspitali University Hospital, Reykjavik, Iceland; 8 Faculty of Medicine and Health, School of Population Health, UNSW Sydney, Sydney, New South Wales, Australia; 9 West China Biomedical Big Data Centre, West China Hospital, Sichuan University, Chengdu, Sichuan, China; 10 Medical Big Data Centre, Sichuan University, Chengdu, Sichuan, China; 11 Department of Epidemiology, Harvard T H Chan School of Public Health, Boston, Massachusetts, USA

**Keywords:** COVID-19, Mental Health, Public Health, trends

## Abstract

**Introduction:**

While changes in the prevalence of depressive symptoms during the COVID-19 pandemic have been described across populations, few studies have incorporated multidimensional variables to characterise the varying effects of the pandemic on the population’s mental health.

**Methods:**

This cohort study included 6423 participants aged ≥18 years from the Icelandic COVID-19 National Resilience Cohort. Data on depressive symptoms and pandemic-related and non-pandemic-related factors were obtained during three pandemic assessment periods (baseline, follow-up wave 1 and follow-up wave 2; April 2020–December 2021), while health outcomes were obtained during the post-pandemic assessment period (follow-up wave 3; September 2022–February 2023). We used latent growth mixture models to identify variation in depressive symptom trajectories during the pandemic. We then used XGBoost models with 37 pandemic-related and non-pandemic-related factors to characterise these trajectories. Moreover, we performed linear regression to assess the association between the identified trajectories and post-pandemic health outcomes.

**Results:**

Of the included participants, we identified four depressive symptom trajectories, including consistently low (83.7%), consistently high (5.3%), initially high (5.1%) and late-onset high (5.9%) symptom trajectories. Individuals who exercised frequently (≥3 days/week) and enjoyed social and family support were more likely to experience a consistently low symptom trajectory. In contrast, individuals with a history of psychiatric disorders, women and young adults (18–39 years) were less likely to follow the consistently low symptom trajectory. Moreover, compared with the consistently low symptom trajectory, the other trajectories were associated with significantly higher levels of depressive, anxiety and somatic symptoms and cognitive problems during the post-pandemic period.

**Conclusions:**

Our results underscore the long-lasting impact of the COVID-19 pandemic on population mental health. Interventions focusing on exercise, social support and family support may mitigate the adverse mental health effects of future pandemics.

**WHAT IS ALREADY KNOWN ON THIS TOPIC**

- The COVID-19 pandemic had a huge impact on people’s daily lives. However, most existing studies have focused on mental health responses during the early stage of the pandemic, and few studies have incorporated multidimensional variables to characterise the depressive symptom trajectories within the general population or examined their post-pandemic health outcomes.

**WHAT THIS STUDY ADDS**

- Leveraging longitudinal data from the Icelandic COVID-19 National Resilience Cohort, our study identified four distinct depressive symptom trajectories during the pandemic period, with 83.7% of persons showing consistently low, 5.3% consistently high, 5.1% initially high and 5.9% late-onset high depressive symptom burden.
- Individuals who exercised frequently (≥3 days/week) and perceived social and family support were more likely to experience a consistently low symptom trajectory during the pandemic, while those with a history of psychiatric disorders, women and young adults (18–39 years) were less likely to follow the consistently low symptom trajectory.
- Compared with the consistently low symptom trajectory, initially high, late-onset high and consistently high symptom trajectories were associated with elevated post-pandemic health outcomes (ie, depressive, anxiety and somatic symptoms and cognitive problems).

**HOW THIS STUDY MIGHT AFFECT RESEARCH, PRACTICE OR POLICY**

- Our study suggests that the COVID-19 pandemic has had a disproportionate impact on the general population, and strategies targeting those most affected (eg, patients with a history of psychiatric disorders, women and young adults) should be prioritised in future pandemics.
- Identified modifiable protective factors, including physical exercise, family support and social support, may mitigate the impact of the pandemic on population mental health and should be recommended at similar times of crisis.

## Introduction

 Mental health and well-being of the general population were severely impacted during the COVID-19 pandemic.^1^ Indeed, a nationally representative survey among US adults suggested that the prevalence of depressive symptoms was more than threefold higher at the beginning of the pandemic when compared with the pre-pandemic period.^2^ A recent meta-regression analysis furthermore suggested that the prevalence of depression in the general population may have increased over time during the pandemic.^3^

Yet, individuals may have experienced different mental health responses during the COVID-19 pandemic and demonstrated heterogeneous mental health trajectories over time.^4 5^ For example, Batterham *et al* identified three latent depressive symptom trajectories among a representative sample of Australian adults,^6^ including low or moderate symptom burden throughout the study period and initially severe followed by declining trajectories. In another sample representative of the adult UK general population,^5^ five distinct mental health trajectories (measured by the 12-item General Health Questionnaire) were identified during the first 6 months of the pandemic (April–September 2020), including consistently poor, deteriorating, recovery, consistently good and consistently very good trajectories. Furthermore, these studies have suggested that younger age,^7^ a history of mental illness^5 6^ and COVID-19-related financial distress^6^ were associated with consistently high depressive symptoms during the pandemic.

However, the findings are inconsistent, and most studies have focused on mental health responses during the early stage of the pandemic.^5^,^7^ In addition, although previous studies have examined the role of sociodemographic factors,^8 9^ lifestyle factors,^10^ quarantine,^11^ severity of COVID-19^12^ and health service disruptions,^13^ few studies have incorporated multidimensional variables to investigate and characterise the varying effects of the pandemic on population mental health. Furthermore, there is a scarcity of follow-up data to explore the long-term outcomes of these trajectories in the post-pandemic period.

To this end, leveraging longitudinal data from the Icelandic COVID-19 National Resilience (C-19 Resilience) Cohort over a period of almost 3 years (ie, from April 2020 to February 2023), we aimed to investigate the variation in depressive symptom trajectories during the COVID-19 pandemic in Iceland, and to further identify the determinants of these varying trajectories as well as the post-pandemic health outcomes associated with each trajectory.

## Materials and methods

### Study design and participants

**Figure 1:**
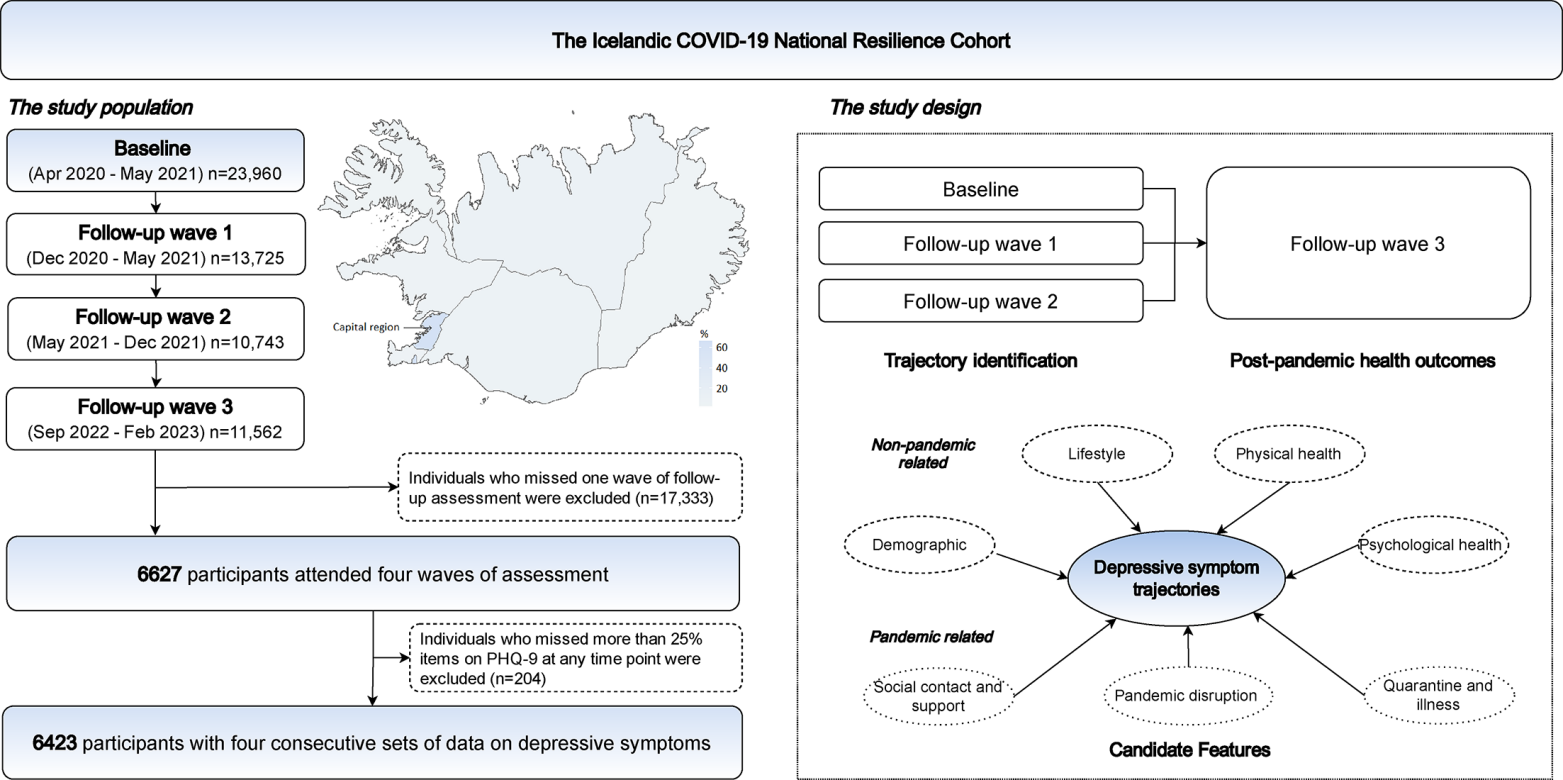
Flowchart outlining the selection of the study population and study design. PHQ-9, Patient Health Questionnaire-9.

We used data from the C-19 Resilience Cohort, which was established in April 2020 and was eligible for all Icelandic-speaking individuals aged ≥18 years in Iceland.^14^ The total population in Iceland aged ≥18 years on 1 January 2020 was 273 190.^15^ Recruitment of the study sample was mainly through social media and invitations to participants in existing nationwide cohort studies, including the Stress-And-Gene-Analysis cohort,^16^ the Iceland Screens, Treats or Prevents Multiple Myeloma study^17^ and the Health and Well-Being of Icelanders cohort.^18^ In total, 23 960 persons were enrolled in the baseline assessment between April 2020 and May 2021 (8.8% of the eligible population), and three waves of follow-up were completed by February 2023 (follow-up wave 1: December 2020–May 2021; follow-up wave 2: May 2021–December 2021; follow-up wave 3: September 2022–February 2023) with the attrition rate ranging from 42.7% to 55.2% (figure 1). As the Icelandic government lifted all pandemic-related social restrictions and border prevention measures by February 2022,^19^ and since 79% of Iceland’s total population were fully vaccinated by April 2022,^20^ we defined the baseline and the first two follow-up waves as the pandemic assessment and the third follow-up wave as the post-pandemic assessment. More COVID-19 information in Iceland is provided in online supplemental sFigure 1.

Participants were invited to complete a series of web-based questionnaires and provide information on demographics, lifestyle and general health, as well as working and life conditions at each assessment wave. In the present study, we included 6423 persons with four consecutive sets of data on depressive symptoms in the analysis. Specifically, we used the first three assessments (ie, baseline, follow-up wave 1 and follow-up wave 2) to explore the variation in depressive symptom trajectories during the pandemic and identify determinants of each trajectory. We then used the latest assessment (ie, follow-up wave 3) to investigate the long-term health outcomes of the identified trajectories after the pandemic. Though largely comparable to participants who were excluded from the study (ie, lost follow-up (n=17 333) or missed information on depressive symptom assessment (n=204)), the study sample was more likely to be older, have a higher level of education and be without childcare burden (online supplemental sTable 1).

### Patient and public involvement

Patients or the public were not involved in the design, or conduct, or reporting, or dissemination plans of our research.

### Measures

#### Depressive symptoms

We used a validated self-report instrument, the Patient Health Questionnaire-9 item (PHQ-9),^21^ to screen for depressive symptoms, over the past 2 weeks. Response options in each item range from ‘0’ (not at all) to ‘3’ (nearly every day), and the total score ranges from 0 to 27. A threshold score of 10 or higher is considered as an indication of high depressive symptom burden.^21^ For participants who responded to more than 75% of items on PHQ-9 at each assessment, we used item-level imputation by predictive mean matching to replace missing values, and then calculated the total score.

#### Candidate features

37 features with potential relevance to depressive symptom trajectories during the pandemic were assessed, including 7 demographic features^5 8 22^ (ie, age, sex, sexual orientation, residence area, relationship status, education and childcare burden), 3 lifestyle features^8 10 23^ (ie, exercise level at baseline, change in exercise habits during the pandemic and smoking status), 4 physical and psychological health features^24^,^27^ (ie, body mass index (BMI), chronic medical conditions, mobility/hearing/visual impairment and history of psychiatric disorders), 10 pandemic-related social contact and support features^28^,^30^ (ie, in-person contact, change in frequency of in-person contact, virtual contact, change in frequency of virtual contact, perceived family support, change in family support, perceived social support, change in social support, trust in Icelandic health authorities and change in trust in the Icelandic health authorities during the pandemic), 7 quarantine-related and COVID-19-related features^11 12 31 32^ (ie, quarantine, COVID-19 testing and diagnosis, bedridden due to COVID-19, family/friends diagnosed with COVID-19, family/friends admitted to a hospital, family/friends admitted to the intensive care unit and vaccination status) and 6 pandemic disruption-related features^13 33^ (ie, financial difficulties, change in financial difficulties, difficulty in obtaining necessities, change in difficulty in obtaining necessities, disruption of necessary services and change in disruption of necessary services during the pandemic).

Changes in specific features during the pandemic were derived by comparing response options from the baseline to those at follow-up wave 2, categorised as decreasing, stable or increasing. Moreover, we used K-nearest neighbour imputation (k=10) to replace missing values of the candidate features (proportions of missing values 0%–2.2%). Online supplemental sTable 2 shows more details about the measuring and scoring rules of the candidate features.

#### Post-pandemic health outcomes

Four validated psychological function instruments were assessed at follow-up wave 3, including depressive symptoms (PHQ-9),^21^ anxiety symptoms (the Generalised Anxiety Disorder 7-item questionnaire, with a total score ranging from 0 to 21),^34^ somatic symptoms (Patient Health Questionnaire-15 (PHQ-15), with a total score ranging from 0 to 30)^35^ and cognitive problems (the 8-item Patient-Reported Outcomes Measurement Information System Cognitive Function Scale, with a total score ranging from 0 to 32).^36^ Item-level imputation by predictive mean matching was used to replace missing items in each instrument. Specifically, considering the PHQ-15 item about menstrual cramps which only applies to women younger than 60 years, we directly imputed 0 (ie, ‘Not bothered at all’) for individuals who were men or older than 60 years with a missing value on this item.

### Statistical analysis

We first examined the demographic features of the study population. Then, we constructed latent growth mixture models using the total score of the PHQ-9 to explore variation in depressive symptom trajectories during the pandemic. Accounting for varying months in which each person responded, we added a TSCORES term in the model to indicate variable time of measurement of depressive symptoms. We then fitted the model from one to seven latent classes. Model fit was determined using the Akaike information criterion (AIC), Bayesian information criterion (BIC), sample-size-adjusted BIC (aBIC) and the Lo-Mendel-Rubin-likelihood ratio test (LMR-LRT).^37^ The model with the lowest AIC, BIC and aBIC was preferred. Significant LMR-LRT results indicate that the specific class solution is more favourable than other classes solutions. In addition to these fit statistics, whether the number of individuals in each trajectory group was sufficient (more than 5.0%) was also considered.^37^ Moreover, the entropy value was calculated to assess the model’s performance, with an entropy near 1.0 indicating adequate classification of individuals. Participants were categorised into probable groups after the selection of the optimal model.

Next, we calculated the Spearman’s rank correlation coefficient (r) among pairs of candidate features and excluded those with very strong correlation with other features (ie, r≥0.8). To identify the most influential features for each trajectory group, we applied the extreme gradient boosting (XGBoost) model with dummy encoding for categorical features and calculated the mean absolute SHapley Additive exPlanation (SHAP) values to rank the feature importance. XGBoost model is an effective tree boosting classifier.^38^ We defined multiple tree-related hyperparameters to improve the model performance and avoid the risk of overfitting, including ntrees (number of trees), colsample_bytree (subsample ratio of columns for each tree), max-depth (maximum depth of a tree), gamma (minimum loss reduction required), minobspernode (minimum observations allowed per tree node) and eta (step size shrinkage).^38^ The SHAP value can quantify the magnitude and direction (positive or negative) of the feature’s contribution to a classification. Namely, a high positive SHAP value indicates a strong positive effect on the classification. To assess the performance of the model, we split the data into training (70%) and test (30%) sets. We used the training set to fit the model and the test set for assessment. Considering the imbalanced group distribution, optimal Youden index and area under the receiver operating curve (AUC) were calculated.

Furthermore, we performed linear regression analyses to explore the associations between depressive symptom trajectories and their post-pandemic health outcomes. We adjusted the analyses for age at baseline, sex, education, relationship status, smoking status, BMI, chronic medical conditions and history of psychiatric disorders, and reported standardised regression coefficients (βs) with corresponding 95% CIs.

The latent growth mixture model and XGBoost model were performed in Mplus V.8.0 and Python V.3.1, respectively. All the other analyses were conducted in R V.4.2 software.

## Results

**Figure 2:**
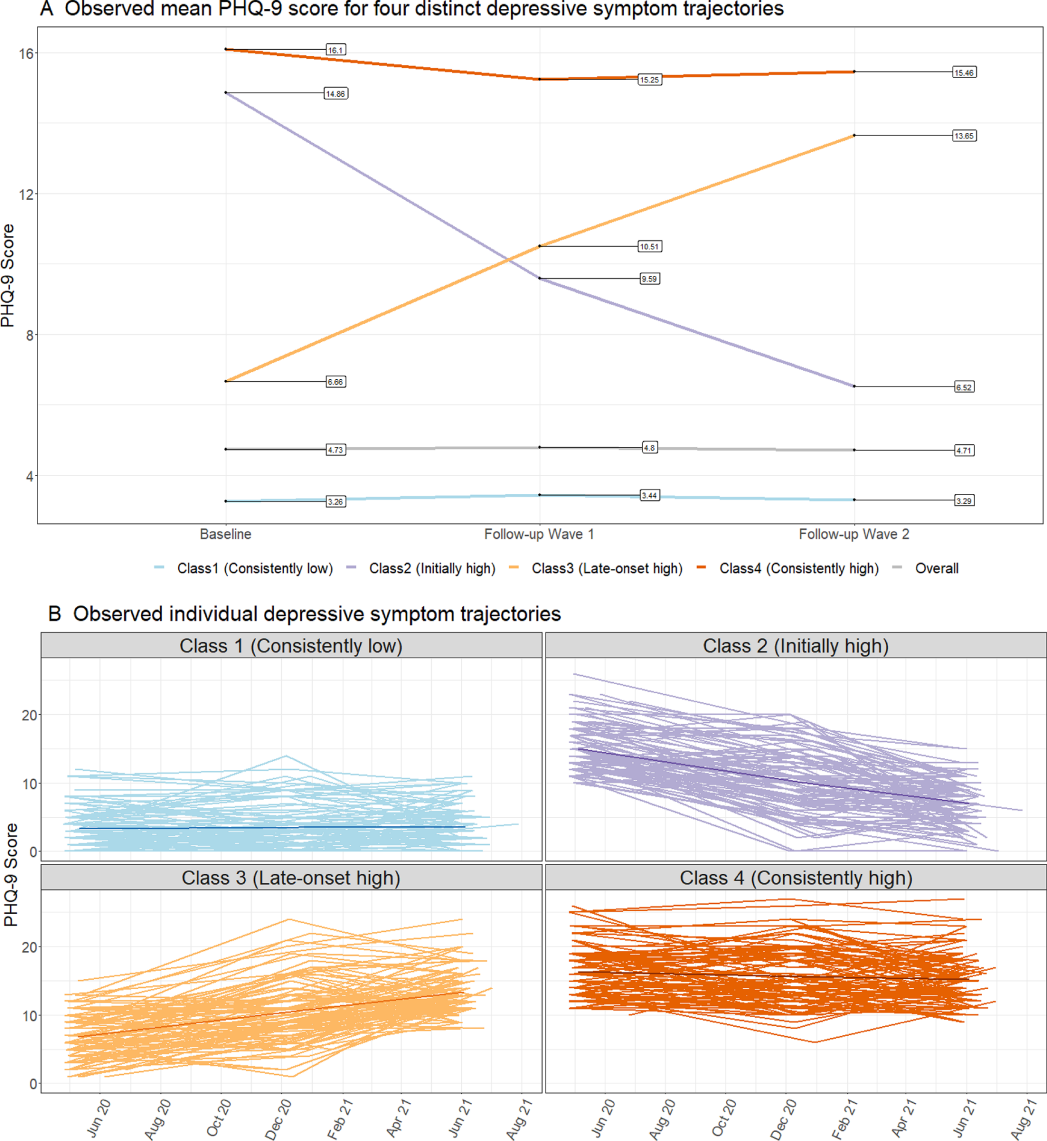
Four class-specific depressive symptom trajectories during the COVID-19 pandemic in Iceland. Note: For panel **B**, we randomly selected a subset of 100 individuals from each class to enhance the clarity of the trajectory. PHQ-9, Patient Health Questionnaire-9.

Of the 6423 participants, 68.7% were women and the mean (SD) age at baseline was 57.0 (13.0) years (online supplemental sTable 1). In the entire sample, the levels of depressive symptom did not change significantly from baseline to follow-up wave 2 (p=0.16; figure 2). After fitting models with 1–7 latent classes, we identified a 4-class model as the ideal model with adequate classification of individuals (ie, significant LMR-LRT results, sufficient number of participants in each class and near 1.0 entropy; online supplemental sTable 3). From this model, we categorised participants into four distinct depressive symptom trajectories.

During a median follow-up of 13.0 months from baseline to follow-up wave 2, we found that most participants (83.7%) showed a trajectory with consistently low depressive symptoms, while 5.3% of individuals showed a trajectory with consistently high symptom burden throughout the study period. In addition, two trajectories with fluctuating depressive symptoms were identified, with 5.1% showing initially high symptom trajectory (ie, high levels of depressive symptoms at baseline which then decreased) and 5.9% showing late-onset high symptom trajectory (ie, low levels of depressive symptoms at baseline which then increased).

**Figure 3:**
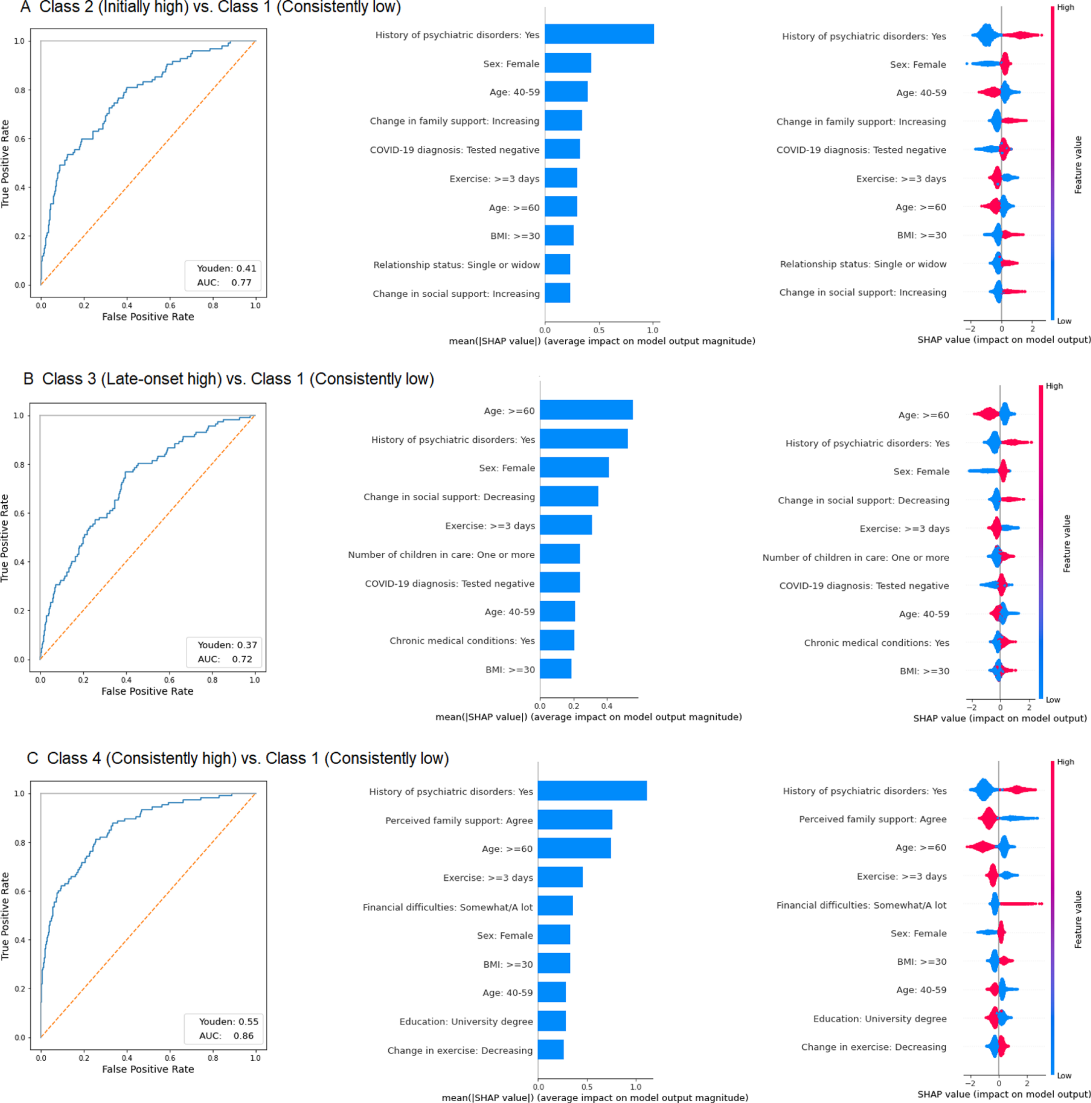
Model performance and 10 most influential features. Youden index, calculated as the maximum of the true positive rate minus the false positive rate. AUC, area under the curve.

A strong correlation between each pair of the candidate features was not observed (online supplemental sFigure 2), and the XGBoost model achieved good performance in distinguishing consistently low symptom trajectory from other trajectories (figure 3; Youden index: 0.37–0.55; AUC: 0.72–0.86). Moreover, individuals who exercised frequently (≥3 days/week) and perceived family support at baseline were more likely to experience the consistently low symptom trajectory during the pandemic. By contrast, those with a history of psychiatric disorders, women and young adults (18–39 years) were less likely to follow consistently low symptom trajectory. In addition, individuals in the initially high symptom trajectory group were characterised by having tested negative for COVID-19 and higher weight (BMI ≥30 kg/m^2^) and single, while people in the late-onset high symptom trajectory group were characterised by having childcare burden, having tested negative for COVID-19 and suffering from chronic medical conditions. Furthermore, compared with consistently low symptom trajectory group, a decrease in the level of social support during the pandemic was associated with a greater risk of showing late-onset high symptom trajectory, while an increase in family support and social support between baseline and follow-up wave 2 was associated with a greater likelihood of experiencing the initially high symptom trajectory.

**Figure 4:**
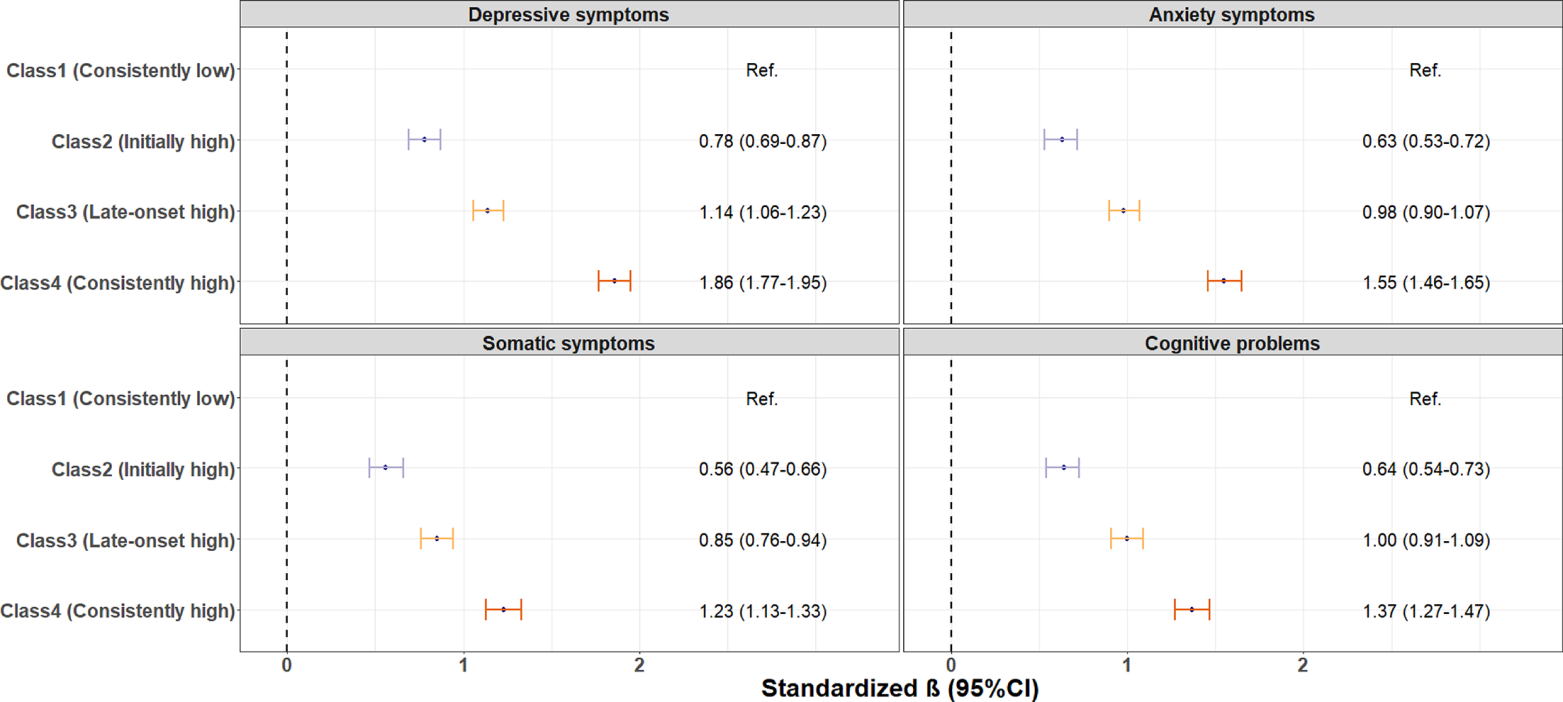
Multivariable-adjusted standard linear regression analyses of the association between depressive symptom trajectories and post-pandemic health outcomes (ie, depressive symptoms, anxiety symptoms, somatic symptoms and cognitive problems measured during follow-up wave 3, from September 2022 to February 2023). Model adjusted for age at baseline, sex, education level, relationship status, smoking status, BMI, chronic medical conditions and history of psychiatric disorder. BMI, body mass index.

After a further median follow-up of 15.8 months from follow-up wave 2 to follow-up wave 3, we observed that individuals who demonstrated initially high, late-onset high and consistently high symptom trajectories during the pandemic were more likely to experience a significantly higher levels of depressive (βs 0.78–1.86, figure 4), anxiety (βs 0.63–1.55) and somatic symptoms (βs 0.56–1.23), as well as cognitive problems (βs 0.64–1.37) during the post-pandemic period, when compared with those in the consistently low symptom trajectory group.

## Discussion

Using a nationwide cohort, we identified four distinct depressive symptom trajectories during the COVID-19 pandemic in Iceland. Though most persons experienced a consistently low symptom trajectory, a minority (16.3%) showed trajectories with high symptom burden at the initial or late pandemic phase, or persistently. The pandemic impact on population mental health seems long-lasting as individuals with these trajectories continued to experience elevated levels of mental illness, somatic symptoms and cognitive problems during the post-pandemic period. Expanding on previous findings, we found that depressive symptoms during the pandemic were associated with a history of psychiatric disorder, being women, being younger (18–39 years) and having tested negative for COVID-19. Furthermore, we also identified several modifiable protective factors, including physical exercise, family support and social support, that may mitigate the impact of the pandemic on population mental health.

Though the main symptom trajectory in the study population was largely constant over time and good, our study identified three additional depressive symptom trajectories during the pandemic period. Compared with the findings of Hemi *et al* in the Israeli population,^7^ which identified two trajectories for depression (resilient, 87% and chronic, 13%), our study indicates a varying and complex development of population mental health in Iceland during the pandemic. However, when compared with the results of a study by Pierce *et al* in the UK population,^5^ we found that a high proportion of the Icelandic general population experienced consistently low symptom trajectories during the pandemic. Several reasons may account for these discrepancies, such as the difference in assessment points, length of follow-up and implemented governmental pandemic policies.^39^ Also, the Icelandic population is small (total population of 400 000)^40^ with close family contacts that may preserve mental health during pandemic times. Finally, the substantially longer follow-up in our study is more likely to reflect the long-term pandemic adaptation trajectories, instead of only the acute responses at the initial stage of the pandemic.

We identified several vulnerability factors during the pandemic, including a history of psychiatric disorder, which, as in other crises^41^ and situations of uncertainty,^42^ may contribute to increased depressive symptoms. Also, reduced access to health services, in particular for patients with a history of psychiatric disorder, may have made these individuals more susceptible to the negative effects of the pandemic.^43^ Indeed, findings from our^13^ and others’^44^ previous work indicate that individuals with pre-pandemic history of psychiatric disorders experienced a disruption in health services during the pandemic. Consistent with existing evidence,^5^ we further found that women and young adults were more likely to experience psychological distress during the pandemic. Potential increase in domestic violence^45^ and greater burden of domestic work and childcare during social gathering restriction^22^ may have contributed to increased depressive symptoms burden among women during the pandemic. The mental health of young adults may have been affected by school and workplace closings and less social contact, as well as economic uncertainties (eg, job loss).^46^ In addition, we found that patients with pre-existing chronic medical conditions faced an elevated risk of developing late-onset depressive symptom trajectory. A possible explanation for the delayed effects could be that individuals with chronic conditions tended to self-isolate in order to reduce their likelihood of contracting COVID-19 initially, but they may have become more vulnerable and depressed as time passes in a pandemic.

Moreover, we found that individuals who tested negative for COVID-19 were more likely to be negatively affected by the pandemic, during either the initial or late pandemic phase. Indeed, previous studies carried out by us^47^ and others^48^ have shown that individuals who tested negative for COVID-19 are associated with an increased risk of psychological distress, as well as receiving prescription for psychotropic medications. Patients who took a screening test but were not diagnosed with COVID-19 might have been exposed to infected individuals and therefore fear infection or have experienced severe influenza symptoms due to other causes which potentially contribute to increased levels of depressive symptoms.^48 49^ It is also possible that participants susceptible to psychiatric disorders or those already suffering from such disorders were more likely to be tested for COVID-19 and therefore showed a high symptom burden of depression.^50^ Thus, a reverse causation cannot be excluded. In addition, unlike previous findings,^5 51^ we did not observe a link between COVID-19 infection and deterioration in mental health. The low prevalence of COVID-19 (ie, 213/6423 (3.3%)) as well as better access to healthcare for COVID-19 patients in the present study population^13^ may be key factors explaining these null results. Taken together, our findings suggest that the COVID-19 pandemic has had a disproportionate impact on different groups of the general population, suggesting that strategies targeting those most affected should be prioritised in future pandemics.

By contrast, we observed that maintaining physical exercise (≥3 days/week) was associated with consistently low depressive symptom trajectories during the pandemic. In line with our findings, a recent review found that physical exercise, especially supervised exercise, was effective in reducing levels of depression during the pandemic, and that the frequency and intensity of the exercise were associated with maintenance of psychological well-being.^52^ Also, the results of a study among students at the University of Pittsburgh by Giuntella *et al* suggested that the disruption in physical activity was a leading risk factor of depression during the pandemic.^10^ Although barriers to increasing activity were likely present, such as the closure of gym facilities and less opportunity to exercise with others, maintaining exercise may be most beneficial in alleviating the population’s psychological distress.^53^ Meanwhile, the positive association between family and social support and mental health is well established.^28 29^ In our study, we indeed observed that an increase in family and social support during the pandemic was associated with a decline in depressive symptoms (ie, initially high symptom trajectory), whereas a decrease in such support was linked to elevated depressive symptoms (ie, late-onset high symptom trajectory). The protective effects of these factors are important for preserving the population’s mental health and should be recommended at similar times of crisis.

### Strengths and limitations

A major strength of this study is the use of a large nationwide cohort to investigate the variation in depressive symptom trajectories during the COVID-19 pandemic in Iceland over a 3-year follow-up period. Moreover, leveraging the wealth of information collected, we were able to incorporate multidimensional variables to thoroughly characterise the risk and protective factors of the identified trajectories. This study also has several limitations. First, due to the lack of pre-pandemic data, our study cannot clearly differentiate between pre-existing depressive symptoms and symptoms that emerged during the pandemic. For example, it is unclear whether depressive symptoms observed at baseline are a result of the pandemic or if they were already present before the pandemic. Second, we allowed within-class variation of individuals in the latent growth mixture models; as such, the identified profiles might not represent all individuals in a specific class. Furthermore, the interpretation of the profiles is subjective, though we followed the guidelines for reporting on latent trajectory studies.^37^ Third, mental health assessments were based on self-report questionnaires rather than clinical diagnostic interviews. In addition, in the setting of the COVID-19 pandemic, several items measured as depressive symptoms (eg, feeling tired, poor appetite, trouble concentrating) in the PHQ-9 instrument may be attributed to COVID-19 infection rather than depression itself. However, the low prevalence of COVID-19 in our study population may suggest a limited impact of this concern. Finally, the recruitment of the study sample was mainly through social media, and the study population was over-represented by older persons, those with higher levels of education and those without childcare burden, which may limit the generalisability of our findings.

## Conclusions

The results of the current study suggest that the vast majority of the Icelandic population maintained good mental health during the COVID-19 pandemic. In addition, our results underscore the role of preexisting psychiatric disorders as a susceptibility factor for experiencing a high level of psychological distress during the pandemic. Interventions focusing on maintaining and enhancing physical exercise and social and family support can help mitigate the negative effects of future pandemics.

## supplementary material

10.1136/bmjph-2024-001294 online supplemental file 1

## Data Availability

No data are available.

## References

[1] Vindegaard N, Benros ME (2020). COVID-19 pandemic and mental health consequences: Systematic review of the current evidence. Brain Behav Immun.

[2] Ettman CK, Abdalla SM, Cohen GH (2020). Prevalence of Depression Symptoms in US Adults Before and During the COVID-19 Pandemic. JAMA Netw Open.

[3] Yuan K, Zheng Y-B, Wang Y-J (2022). A systematic review and meta-analysis on prevalence of and risk factors associated with depression, anxiety and insomnia in infectious diseases, including COVID-19: a call to action. Mol Psychiatry.

[4] Kimhi S, Eshel Y, Marciano H (2021). Trajectories of depression and anxiety during COVID-19 associations with religion, income, and economic difficulties. J Psychiatr Res.

[5] Pierce M, McManus S, Hope H (2021). Mental health responses to the COVID-19 pandemic: a latent class trajectory analysis using longitudinal UK data. Lancet Psychiatry.

[6] Batterham PJ, Calear AL, McCallum SM (2021). Trajectories of depression and anxiety symptoms during the COVID-19 pandemic in a representative Australian adult cohort. Med J Aust.

[7] Hemi A, Sopp MR, Bonanno G (2024). Flexibility predicts chronic anxiety and depression during the first year of the COVID-19 pandemic-A longitudinal investigation of mental health trajectories. Psychol Trauma.

[8] Cook S, Saburova L, Bobrova N (2021). Socio-demographic, behavioural and psycho-social factors associated with depression in two Russian cities. J Affect Disord.

[9] Hawes MT, Szenczy AK, Klein DN (2022). Increases in depression and anxiety symptoms in adolescents and young adults during the COVID-19 pandemic. Psychol Med.

[10] Giuntella O, Hyde K, Saccardo S (2021). Lifestyle and mental health disruptions during COVID-19. Proc Natl Acad Sci USA.

[11] Brooks SK, Webster RK, Smith LE (2020). The psychological impact of quarantine and how to reduce it: rapid review of the evidence. The Lancet.

[12] Magnúsdóttir I, Lovik A, Unnarsdóttir AB (2022). Acute COVID-19 severity and mental health morbidity trajectories in patient populations of six nations: an observational study. Lancet Public Health.

[13] Wang Y, Unnarsdóttir AB, Magnúsdóttir I (2024). Trends of perceived disruption in healthcare services during the pandemic: findings from the COVID-19 National Resilience Cohort in Iceland. Eur J Public Health.

[14] Unnarsdóttir AB, Lovik A, Fawns-Ritchie C (2022). Cohort Profile: COVIDMENT: COVID-19 cohorts on mental health across six nations. Int J Epidemiol.

[15] Statistics Iceland Average population by sex and age 1841-2023. https://px.hagstofa.is/pxen/pxweb/en/Ibuar/Ibuar__mannfjoldi__1_yfirlit__yfirlit_mannfjolda/MAN08000.px/table/tableViewLayout2/2024).

[16] The saga cohort.

[17] Rögnvaldsson S, Love TJ, Thorsteinsdottir S (2021). Iceland screens, treats, or prevents multiple myeloma (iStopMM): a population-based screening study for monoclonal gammopathy of undetermined significance and randomized controlled trial of follow-up strategies. Blood Cancer J.

[18] Directorate of Health (2023). Health and wellbeing. https://island.is/en/health-and-wellbeing.

[19] Government of Iceland (2022). COVID-19: Lifting of All Domestic Restrictions and Restrictions at the Border, Ministry of Health MfFA, Editor.

[20] Yelena (2022). What’s the status of covid-19 vaccination in Iceland?. https://www.icelandreview.com/ask-ir/whats-the-status-of-covid-19-vaccination-in-iceland/2024.

[21] Levis B, Benedetti A, Thombs BD (2019). Accuracy of Patient Health Questionnaire-9 (PHQ-9) for screening to detect major depression: individual participant data meta-analysis. BMJ.

[22] Sevilla A, Smith S (2020). Baby steps: the gender division of childcare during the COVID-19 pandemic. Oxford Rev Econ Policy.

[23] Steptoe A, Di Gessa G (2021). Mental health and social interactions of older people with physical disabilities in England during the COVID-19 pandemic: a longitudinal cohort study. Lancet Public Health.

[24] Luppino FS, de Wit LM, Bouvy PF (2010). Overweight, obesity, and depression: a systematic review and meta-analysis of longitudinal studies. Arch Gen Psychiatry.

[25] Robillard R, Daros AR, Phillips JL (2021). Emerging New Psychiatric Symptoms and the Worsening of Pre-existing Mental Disorders during the COVID-19 Pandemic: A Canadian Multisite Study: Nouveaux symptômes psychiatriques émergents et détérioration des troubles mentaux préexistants durant la pandémie de la COVID-19: une étude canadienne multisite. Can J Psychiatry.

[26] Na L, Yang L (2022). Psychological and behavioral responses during the COVID-19 pandemic among individuals with mobility and/or self-care disabilities. Disabil Health J.

[27] Castro-de-Araujo LFS, Rodrigues E da S, Machado DB (2022). Multimorbidity worsened anxiety and depression symptoms during the COVID-19 pandemic in Brazil. J Affect Disord.

[28] Ozbay F, Johnson DC, Dimoulas E (2007). Social support and resilience to stress: from neurobiology to clinical practice. Psychiatry (Edgmont).

[29] Li S, Xu Q (2022). Family support as a protective factor for attitudes toward social distancing and in preserving positive mental health during the COVID-19 pandemic. J Health Psychol.

[30] Sommerlad A, Marston L, Huntley J (2022). Social relationships and depression during the COVID-19 lockdown: longitudinal analysis of the COVID-19 Social Study. Psychol Med.

[31] Lovik A, González-Hijón J, Hoffart A (2023). Elevated symptoms of depression and anxiety among family members and friends of critically ill COVID-19 patients - an observational study of five cohorts across four countries. Lancet Reg Health Eur.

[32] Nguyen M (2021). The Psychological Benefits of COVID-19 Vaccination. Adv Public Health.

[33] Fu M, Guo J, Zhang Q (2023). The associations of pandemic-related difficulties with depressive symptoms and psychological growth among American older adults: Social support as moderators. J Health Psychol.

[34] Spitzer RL, Kroenke K, Williams JBW (2006). A brief measure for assessing generalized anxiety disorder: the GAD-7. Arch Intern Med.

[35] Kocalevent RD, Hinz A, Brähler E (2013). Standardization of a screening instrument (PHQ-15) for somatization syndromes in the general population. BMC Psychiatry.

[36] Iverson GL, Marsh JM, Connors EJ (2021). Normative Reference Values, Reliability, and Item-Level Symptom Endorsement for the PROMIS® v2.0 Cognitive Function-Short Forms 4a, 6a and 8a. Arch Clin Neuropsychol.

[37] van de Schoot R, Sijbrandij M, Winter SD (2017). The GRoLTS-Checklist: Guidelines for Reporting on Latent Trajectory Studies. Struct Equ Modeling.

[38] Chen T, Guestrin C (2016). Xgboost: a scalable tree boosting system.

[39] Hale T, Angrist N, Goldszmidt R (2021). A global panel database of pandemic policies (Oxford COVID-19 Government Response Tracker). Nat Hum Behav.

[40] Registers Iceland The people of iceland. https://www.skra.is/english/2024.

[41] Sullivan G, Vasterling JJ, Han X (2013). Preexisting mental illness and risk for developing a new disorder after hurricane Katrina. J Nerv Ment Dis.

[42] Carleton RN, Mulvogue MK, Thibodeau MA (2012). Increasingly certain about uncertainty: Intolerance of uncertainty across anxiety and depression. J Anxiety Disord.

[43] Taxiarchi VP, Senior M, Ashcroft DM (2023). Changes to healthcare utilisation and symptoms for common mental health problems over the first 21 months of the COVID-19 pandemic: parallel analyses of electronic health records and survey data in England. Lancet Reg Health Eur.

[44] Di Gessa G, Maddock J, Green MJ (2022). Pre-pandemic mental health and disruptions to healthcare, economic and housing outcomes during the COVID-19 pandemic: evidence from 12 UK longitudinal studies. Br J Psychiatry.

[45] Stripe N (2020). Domestic Abuse in England and Wales Overview: November 2020.

[46] Benzeval M, Borkowska M, Burton J (2020). Understanding society COVID-19 survey April briefing note: Home schooling. Unders Soc Work Pap.

[47] Wang Y, Ge F, Wang J (2023). Trends in incident diagnoses and drug prescriptions for anxiety and depression during the COVID-19 pandemic: an 18-month follow-up study based on the UK Biobank. Transl Psychiatry.

[48] Abel KM, Carr MJ, Ashcroft DM (2021). Association of SARS-CoV-2 Infection With Psychological Distress, Psychotropic Prescribing, Fatigue, and Sleep Problems Among UK Primary Care Patients. *JAMA Netw Open*.

[49] Li G, Zhou J, Yang G (2021). The Impact of Intolerance of Uncertainty on Test Anxiety: Student Athletes During the COVID-19 Pandemic. Front Psychol.

[50] van der Meer D, Pinzón-Espinosa J, Lin BD (2020). Associations between psychiatric disorders, COVID-19 testing probability and COVID-19 testing results: findings from a population-based study. BJPsych Open.

[51] Thompson EJ, Stafford J, Moltrecht B (2022). Psychological distress, depression, anxiety, and life satisfaction following COVID-19 infection: evidence from 11 UK longitudinal population studies. Lancet Psychiatry.

[52] Ai X, Yang J, Lin Z (2021). Mental Health and the Role of Physical Activity During the COVID-19 Pandemic. Front Psychol.

[53] Diamond R, Waite F (2021). Physical activity in a pandemic: A new treatment target for psychological therapy. *Psychol Psychother*.

