# Supplementary material for: Depressive symptom trajectories among general population during the COVID-19 pandemic in Iceland: a prospective cohort study (2020–2023)
Source: BMJ Public Health. 2024 Nov 4;2(2):e001294. doi: 10.1136/bmjph-2024-001294 (PMC11816283; doi:10.1136/bmjph-2024-001294)

Supplementary Material

sTable 1 Demographic characteristics of the study population.

	**Participants included in the study** (n=6,423)	**Participants excluded from the study** (n=17,537)	**C-19 Resilience Cohort** (n=23,960)
**Age at baseline, years**			
Mean (SD)	57.0 (13.0)	53.1 (14.7)	54.1 (14.4)
Median [Min, Max]	58.0 [18.0, 89.0]	55.0 [18.0, 100]	56.0 [18.0, 100]
**Age group, years**			
18-39	711 (11.1%)	3367 (19.2%)	4078 (17.0%)
40-59	2715 (42.3%)	7688 (43.8%)	10403 (43.4%)
≥60	2997 (46.7%)	6482 (37.0%)	9479 (39.6%)
**Sex**			
Male	1998 (31.1%)	5016 (28.6%)	7014 (29.3%)
Female	4414 (68.7%)	11848 (67.6%)	16262 (67.9%)
Unknown	11 (0.2%)	673 (3.8%)	684 (2.9%)
**Sexual orientation**			
Heterosexual	6156 (95.8%)	16166 (92.2%)	22322 (93.2%)
Sexual minorities	238 (3.7%)	632 (3.6%)	870 (3.6%)
Unknown	29 (0.5%)	739 (4.2%)	768 (3.2%)
**Residence area**			
Not capital area	4391 (68.4%)	11553 (65.9%)	15944 (66.5%)
In capital area	1998 (31.1%)	5222 (29.8%)	7220 (30.1%)
Unknown	34 (0.5%)	762 (4.3%)	796 (3.3%)
**Relationship status**			
Married or in a relationship	4797 (74.7%)	13009 (74.2%)	17806 (74.3%)
Single or widow	1606 (25.0%)	3817 (21.8%)	5423 (22.6%)
Unknown	20 (0.3%)	711 (4.1%)	731 (3.1%)
**Education**			
Primary school	830 (12.9%)	2546 (14.5%)	3376 (14.1%)
High school	1928 (30.0%)	5307 (30.3%)	7235 (30.2%)
University degree	3629 (56.5%)	8930 (50.9%)	12559 (52.4%)
Unknown	36 (0.6%)	754 (4.3%)	790 (3.3%)
**Childcare burden (i.e., number of children in need of care)**			
None	4394 (68.4%)	10365 (59.1%)	14759 (61.6%)
One or more	2000 (31.1%)	6486 (37.0%)	8486 (35.4%)
Unknown	29 (0.5%)	686 (3.9%)	715 (3.0%)

sTable 2 Features used to identify variation in depressive symptom trajectories.

Variables	Data collection	Response options
**Non-pandemic related**		
**Demographic**		
Age at baseline (years)	Baseline	0: 18-39; 1: 40-59; 2: 60+
Sex	Baseline	0: Male; 1: Female
Sexual orientation	Baseline	0: Heterosexual; 1: Sexual minorities
Residence area	Baseline	0: Not capital area; 1: In capital area
Relationship status	Baseline	0: Married or in a relationship; 1: Single or widow
Education	Baseline	0: Primary school; 1: High school; 2: University degree
Childcare burden (i.e., number of children in need of care)	Baseline	0: None; 1: One or more
**Lifestyle**		
Exercise *(i.e., In the past 7 days, how many days have you engaged in vigorous exercise for a total of 30 minutes or more per day?)*	Baseline	0: 0-2 days/week; 1: ≥3 days/week
Change in exercise	Baseline; Follow-up Wave 2	0: Decreasing; 1: Stable; 2: Increasing
Smoking status	Baseline	0: Never; 1: Current/Previous
**Physical and psychological health**		
Body mass index (kg/m^2)	Baseline	0: ≤25; 1: 25-30; 2: ≥30
Chronic medical conditions[a]	Baseline	0: No; 1: Yes
Mobility/hearing/visual impairment	Baseline	0: No; 1: Yes
History of psychiatric disorders	Baseline	0: No; 1: Yes
**Pandemic related**		
**Social contact and support**		
In-person contact *(i.e., meet family, friends, or other people in your home or elsewhere)*	Baseline	0: Never; 1: Sometimes; 2: Often
Change in frequency of in-person contact	Baseline; Follow-up Wave 2	0: Decreasing; 1: Stable; 2: Increasing
Virtual contact *(i.e., in contact with family, friends, or other people through the phone or social media)*	Baseline	0: Never; 1: Sometimes; 2: Often
Change in frequency of virtual contact	Baseline; Follow-up Wave 2	0: Decreasing; 1: Stable; 2: Increasing
Perceived family support *(i.e., get the emotional help and support I need from my family)*	Baseline	0: Disagree; 1: Neutral; 2: Agree
Change in family support	Baseline; Follow-up Wave 2	0: Decreasing; 1: Stable; 2: Increasing
Perceived social support *(i.e., have a special person who is a real source of comfort to me)*	Baseline	0: Disagree; 1: Neutral; 2: Agree
Change in social support	Baseline; Follow-up Wave 2	0: Decreasing; 1: Stable; 2: Increasing
Trust in Icelandic health authorities	Baseline	0: Little; 1: Somewhat; 2: A lot
Change in trust	Baseline; Follow-up Wave 2	0: Decreasing; 1: Stable; 2: Increasing
**Quarantine and illness**		
Quarantine	Baseline; Follow-up Wave 1 & 2	0: No; 1: Yes
COVID-19 testing and diagnosis	Baseline; Follow-up Wave 1 & 2	0: No tested for COVID-19; 1: Tested negative for COVID-19 2: Tested positive for COVID-19.
Bedridden due to COVID-19	Baseline; Follow-up Wave 1 & 2	0: No; 1: Yes
Family/friends diagnosed with COVID-19	Baseline; Follow-up Wave 1 & 2	0: No; 1: Yes
Family/friends admitted to a hospital	Baseline; Follow-up Wave 1 & 2	0: No; 1: Yes
Family/friends admitted to ICU	Baseline; Follow-up Wave 1 & 2	0: No; 1: Yes
Vaccination status	Follow-up Wave 2	0: No; 1: Yes
**Pandemic disruption**		
Financial difficulties	Baseline	0: Little; 1: Somewhat; 2: A lot
Change in financial difficulties	Baseline; Follow-up Wave 2	0: Decreasing; 1: Stable; 2: Increasing

Difficulty obtaining necessities	Baseline	0: Little; 1: Somewhat; 2: A lot
Change in difficulty obtaining necessities	Baseline; Follow-up Wave 2	0: Decreasing; 1: Stable; 2: Increasing
Disruption of necessary services	Baseline	0: Little; 1: Somewhat; 2: A lot
Change in disruption of necessary services	Baseline; Follow-up Wave 2	0: Decreasing; 1: Stable; 2: Increasing

a. Chronic medical conditions were defined as high blood pressure, heart disease, lung disease, chronic kidney disease, cancer, diabetes, immunosuppressive state or immunosuppressive therapy.

b. COVID-19 testing and diagnosis was defined according to responses to questions “Have you been tested for COVID-19?” and “Have you been diagnosed with the COVID-19?”

sTable 3 Model fit statistics for depressive symptom trajectories by different class solutions

	Log-Likelihood	AIC	BIC	Adj-BIC	LMR-LRT p-value	Entropy	individuals per class						
1-class	-51242.2	102500.3	102554.5	102529.0	-	-	100.0%						
2-Class	-50283.4	100588.8	100663.3	100628.3	0.00	0.91	89.1%	10.9%					
3-Class	-49777.7	99583.4	99678.1	99633.6	0.00	0.91	85.3%	8.4%	6.3%				
**4-Class**	**-49439.2**	**98912.3**	**99027.4**	**98973.4**	**0.00**	**0.92**	**83.7%**	**5.9%**	**5.3%**	**5.1%**			
5-Class	-49205.6	98451.1	98586.5	98522.9	0.00	0.89	75.1%	12.8%	6.4%	3.6%	2.2%		
6-Class	-49056.4	98158.8	98314.4	98241.4	0.04	0.90	75.4%	10.9%	6.1%	3.0%	2.7%	2.0%	
7-Class	-48929.8	97911.5	98087.5	98004.9	0.05	0.87	67.2%	16.8%	4.9%	4.3%	3.6%	2.6%	0.7%

Abbreviations: AIC, Akaike information criterion; BIC, Bayesian information criterion; Adj-BIC, adjusted bayesian information criterion; LMR-LRT, Lo–Mendel–Rubin–likelihood ratio test.

sFigure 1 Timeline of data collections.

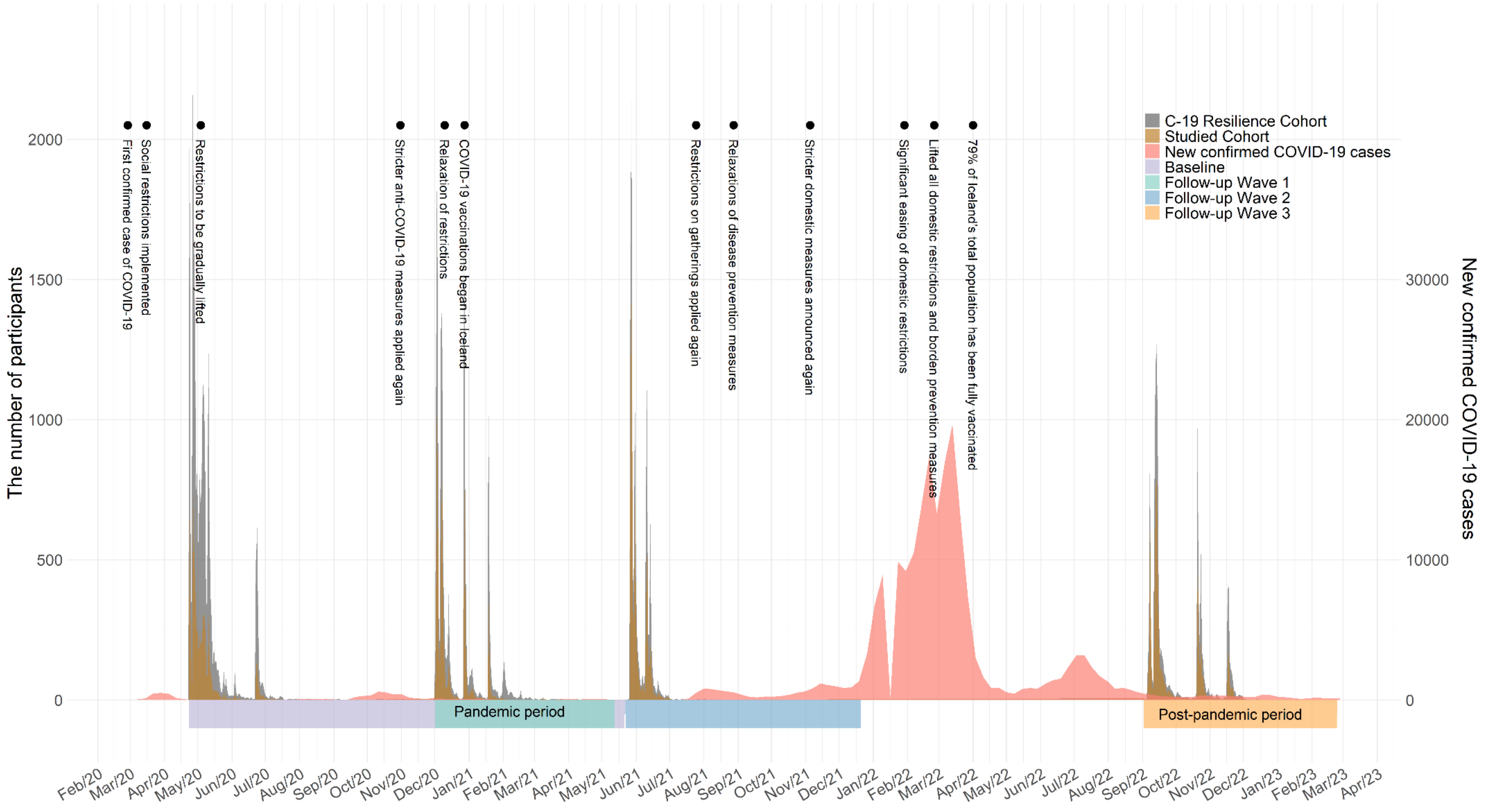

sFigure 2 Spearman's rank correlation matrix of 37 candidate features

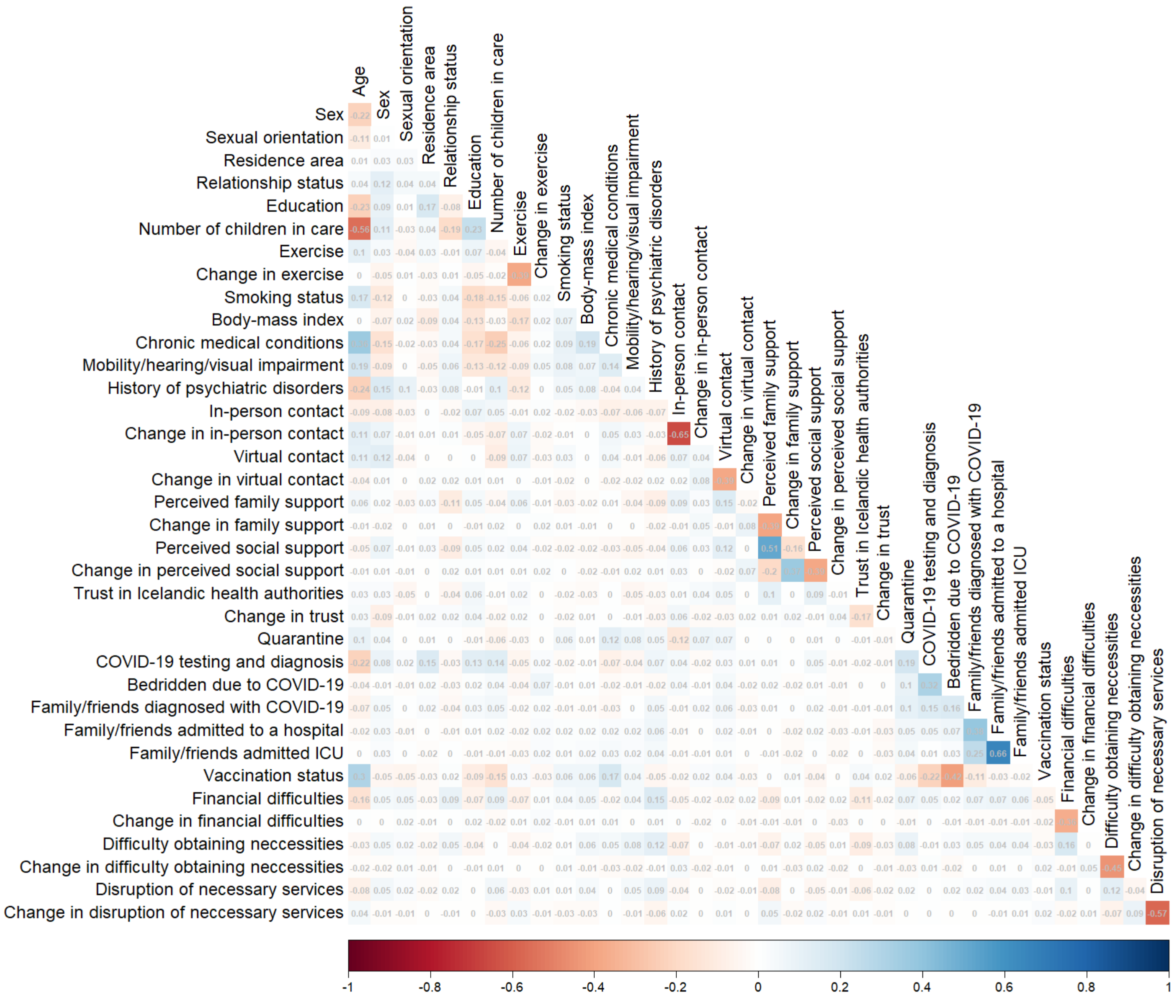

Supplement: online supplemental file 1 [file bmjph-2-2-s001.pdf]
